# The effect of the antibiotic stewardship program (ASP) on community-acquired pneumonia (CAP): a before–after study

**DOI:** 10.3389/fphar.2024.1406960

**Published:** 2024-08-06

**Authors:** Adina Fésüs, Phiona Baluku, Éva Sipos, Sándor Somodi, Enikő Berczi-Kun, István Lekli, Ildikó Bácskay, Ria Benkő, Attila Vaskó

**Affiliations:** ^1^ Department of Pharmacology, Faculty of Pharmacy, University of Debrecen, Debrecen, Hungary; ^2^ Complex Multidisciplinary Health Industry Competence Centre at the University of Debrecen, Debrecen, Hungary; ^3^ Department of Emergency Care and Oxyology, University of Debrecen, Debrecen, Hungary; ^4^ Department of Pharmaceutical Technology, Faculty of Pharmacy, University of Debrecen, Debrecen, Hungary; ^5^ Central Pharmacy, Albert Szent Györgyi Medical Centre, University of Szeged, Szeged, Hungary; ^6^ Clinical Pharmacy Department, Faculty of Pharmacy, University of Szeged, Szeged, Hungary; ^7^ Department of Pulmonology, Faculty of Medicine, University of Debrecen, Debrecen, Hungary

**Keywords:** community-acquired pneumonia, hospitalized patients, empirical antibiotic therapy, antibiotic stewardship, clinical outcomes, intervention

## Abstract

**Background:** Community-acquired pneumonia (CAP) is one of the leading causes of death worldwide. Antibiotic stewardship program (ASP) has been implemented to improve rational and responsible antibiotic use by encouraging guideline adherence.

**Objective:** This retrospective observational before–after study aimed to evaluate whether the ASP may improve guideline adherence, antibiotic exposure, and clinical outcomes in patients hospitalized due to CAP in Hungary.

**Methods:** The study was conducted at a pulmonology department of a tertiary care medical center in Hungary. The ASP implementation consisted of written and published guidelines available to all professionals, continuous supervision, and counseling services on antibiotic therapies at an individual level, with the aim of ensuring compliance with CAP guidelines. Overall guideline adherence (agent selection, route of administration, and dose), clinical outcomes (length of stay and 30-day mortality), antibiotic exposure, and direct costs were compared between the two periods. Fisher’s exact test and *t*-test were applied to compare categorical and continuous variables, respectively. *P*-values below 0.05 were defined as significant.

**Results:** Significant improvement in overall CAP guideline adherence (30.2%), sequential therapy (10.5%), and a significant reduction in the total duration of antibiotic therapy (13.5%) were observed. Guideline non-adherent combination therapies with metronidazole decreased significantly by 28.1%. Antibiotic exposure decreased by 7.2%, leading to a significant decrease in direct costs (23.6%). Moreover, the ASP had benefits for clinical outcomes, and length of stay decreased by 13.5%.

**Conclusion:** The ASP may play an important role in optimizing empirical antibiotic therapy in CAP having a sustained long-term effect.

## Introduction

The use of antibiotics is a cornerstone for the causal treatment of bacterial pneumonia, especially of community-acquired pneumonia (CAP), one of the most common infectious diseases requiring hospitalization (Kosar et al., 2017).

Mortality and morbidity due to antibiotic resistance have increased significantly in recent years (Antimicrobial Resistance et al., 2022). Although the use of antibacterial agents has significantly reduced CAP-related mortality, their inappropriate use has led to the emergence of antibiotic resistance (Lopez-Lozano et al., 2019; Ghosh et al., 2020). Consequently, inappropriately treated CAP may be associated with prolonged hospital stay, placing a heavy financial burden on the healthcare system (Fine et al., 2000; Luthi-Corridori et al., 2023). In 2020 in Hungary, standardized death rates for pneumonia were 8.9 per 100,000 inhabitants for women and 17.1 per 100,000 inhabitants for men. However, these rates included deaths caused by COVID pneumonia (Eurostat, 2024). Moreover, between 2016 and 2021, the average length of stay for pneumonia among in-patients decreased from 12.2 to 10.9 days (Eurostat, 2024). In our country, based on the latest surveillance data on the antimicrobial consumption of the ECDC (European Center for Disease Prevention and Control), the total antimicrobial consumption (community and hospital sector) was 14.4 DDD/1000 inhabitants/day, out of which 1.04 DDD/1000 inhabitants/day indicates the use of antibiotics in the hospital sector (Control, 2023). Furthermore, in the last 10 years, based on the ECDC surveillance report, there has been no decrease in antibiotic consumption in the Hungarian hospital care sector. In fact, a marked increase could be observed in the proportional use of reserve antibiotics used for the treatment of confirmed or suspected infections due to multidrug resistant organisms (Benko et al., 2022; Control, 2024; EC, 2024; ECDC, 2024).

At the same time, prescribing antibiotic treatment has become a major health challenge worldwide. The antibiotic stewardship program (ASP) is implemented to improve the rational and responsible use of antibiotics to improve disease outcomes and reduce antibiotic resistance, healthcare-related infections, and healthcare costs (Tiri et al., 2020). However, the appropriate ASP can only be planned after the identification of antibiotic treatment practices (choice of agent, dosage, dosage form, and duration) (Szalka, 2013; Tiri et al., 2020).

The use of the ASP resulted in significantly lower antibiotic exposure, decreased inappropriate antibiotic use, and limited unintended consequences such as antimicrobial resistance development (Garau et al., 2014). According to a multicenter controlled before-and-after study in Denmark, the ASP led to significantly lower antibiotic exposure and a higher guideline-adherent empirical antibiotic exposure, but without reduction in intravenous therapy (Fally et al., 2021). Another pre–post-intervention study conducted in a pediatric community healthcare center in Israel shows that the ASP resulted in a reduction in broad-spectrum antibiotic use and an increase in guideline-adherent treatment of CAP (Cohen et al., 2022).

To our knowledge, there has been no published official national ASP strategy yet in Hungary. Nevertheless, at the department of pulmonology, a local ASP was implemented with the aim to slow the emergence of antibiotic resistance and optimize antibiotic use. The aims of this study were to evaluate the impact of ASP on guideline adherence in relation to antibiotic selection, route of administration, dose and duration, antibiotic exposure, and costs, as well as clinical outcomes in CAP.

## Materials and methods

### Study design

This was a single-center retrospective observational before–after study managed at the pulmonology department of a tertiary care center in Hungary. At the tertiary care center level, an ASP (titled antibiotic stewardship pilot project) was implemented in June 2019 with the aim of controlling antibiotic use. Restricted antibiotics were listed, which were allowed to be prescribed by physicians and dispensed by the clinical pharmacist only with the permission of infectious disease specialists. Furthermore, written local guidelines for CAP were available at the wards in the pulmonology department, adherence to which was not mandatory but strongly recommended. The COVID pandemic interrupted the ASP, which was resumed in June 2022. However, written guidelines remained available even after the pandemic (10 January 2021). Consequently, data collection in the pre-intervention phase was carried out from 1 January to 30 April 2022, while in the ASP phase, it was from 1 January to 31 March 2023, months with the highest number of CAP cases. Ethics approval was obtained from the Regional Institutional Research Ethics Committee, Clinical Center, University of Debrecen (DE RKEB/IKEB: 6267-2022).

### ASP implementation

**FIGURE 1:**
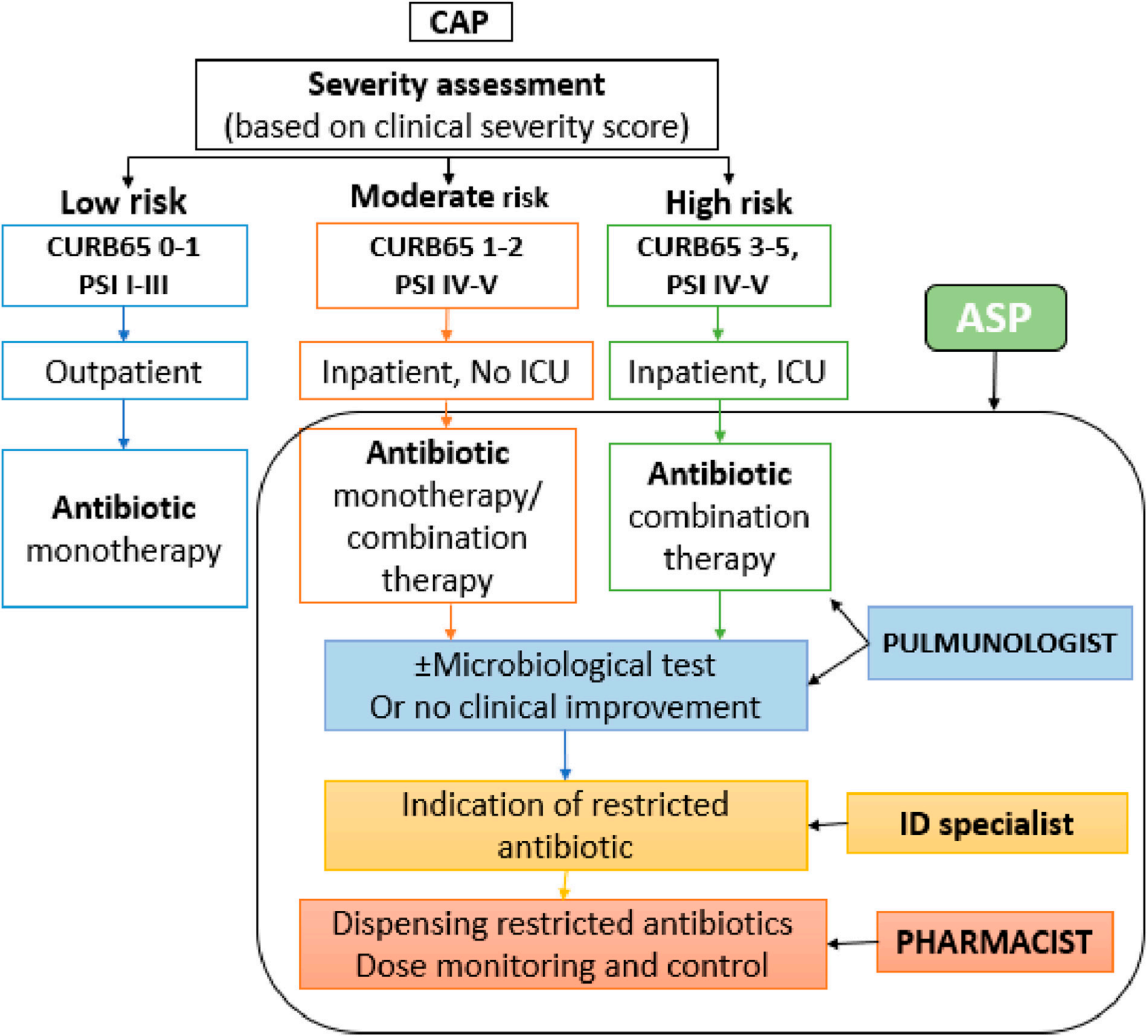
Implemented ASP. CAP, community-acquired pneumonia; CURB-65, confusion, uremia, respiratory rate, blood pressure, (age ≥65 years) score; PSI, Pneumonia Severity Index; ICU, intensive care unit; ID: infectious diseases.

The ASP was introduced in all inpatient care units of the aforementioned center in order to plan the analysis and control of the reasonable and cost-effective use of antibiotics (Center, 2022). The ASP was carried out by the AST (antimicrobial stewardship team) interdisciplinary team consisting of physician specialists (in this study pulmonologists), microbiologists, infectious disease specialists, and pharmacists. The ASP instructions included antibiotic protocols (prophylaxis and empirical therapy), restricted (controlled) antibiotic agents, individual regulation of antibiotic use by infectious disease specialists, elements of infection control, and analysis of antibiotic use. Furthermore, the ASP-guided empirical antibiotic therapy, which was developed based on the local microbiological resistance map and evidence-based antibiotic use. According to these criteria, the narrowest-spectrum agent was recommended in an appropriate dose, adapted to the location and type of infection, for the shortest possible therapeutic period, and in the most optimal route of administration, preferably in monotherapy. Moreover, to reduce the risk of spreading resistance and ensure prudent use, a restriction on antibiotics has been introduced. Restricted antibiotics are as follows: ciprofloxacin, levofloxacin, moxifloxacin, cefiderocol, ceftaroline, ceftazidime/avibactam, ceftolozane/tazobactam, imipenem/cilastatin, meropenem, imipenem/cilastatin/relebactam, meropenem/vaborbactam, linezolid, tedizolid, aztreonam, and colistin*.* The use of these antibiotics was allowed only after infectious disease specialist approval. An electronic approval request form was filled by the pulmonologist and sent to the infectious disease specialist. Required data consisted of patient data, required agent (based on positive microbiological test results—pathogen and sensitivity—or pulmonologist decision when no microbiological test is available and no clinical improvement), first empirical antibiotic therapy, present clinical outcomes, and pulmonologist contact information. Restricted antibiotics could have been dispensed only by the clinical pharmacist under strict control after the approval of the consultant infectologist (Figure 1).

To predict 30-day mortality in CAP, CURB-65 (NICE—National Institute for Health and Care Excellence guideline) score and PSI (Pneumonia Severity Index) were used and determined (Fine, 2023; Macfarlane, 2023). According to the local guideline, patients with CURB65 score 1–5 or PSI IV–V mostly required hospitalization. The local guideline for CAP for inpatients is presented in Table 1.

**TABLE 1:** Guideline-adherent empirical antibiotic therapy in this study (Debrecen, 2023).

Antibiotic	In-patient	
	Moderate risk of mortality CURB-65: 1–2	High risk of mortality CURB-65: 3–5
Monotherapy		
*Suspected typical pathogens*		
Amoxicillin/amoxicillin–clavulanate^a^	✓	
Cefuroxime^b^	✓	
Ceftriaxone^a^	✓	
Cefotaxime^a^	✓	
*Suspected atypical pathogen*		
Doxycycline^a^	✓	
*Severe beta-lactam allergy*		
Moxifloxacin and levofloxacin	✓✓	✓✓
Combination therapy		
Amoxicillin–clavulanate/ampicillin–sulbactam/ceftriaxone/cefotaxime^a^ + clarithromycin/azithromycin/doxycycline		✓
Cefuroxime^b^ + clarithromycin/azithromycin/doxycycline		✓
Piperacillin–tazobactam/cefepime/ceftazidime^c^ + clarithromycin/azithromycin		✓
Meropenem/imipenem or ceftaroline^b^ + clarithromycin/azithromycin		✓✓
Days	Minimum 5	Minimum 5–7

^a^ First-line antibiotic when severity of CAP and comorbidities indicate hospitalization.

^b^ Antibiotics considered during winter in the viral epidemic period.

^c^ Severe CAP, often requiring intensive care; ✓, guideline adherent therapy, no need for infectious disease specialist approval; ✓✓, guideline adherent therapy with restricted antibiotics, need for infectologist approval.

Based on the local guidelines, the recommended systemic antibiotic for moderate risk of mortality was mainly monotherapy, while combination therapy was recommended mainly for high risk of mortality (Debrecen, 2023). Empirical antibiotic treatment was recommended to start within 4 to 6 hours after hospital admission. According to the route of administration, starting therapy with intravenous administration was recommended. Switching from intravenous to oral therapy should be considered after clinical stability, in the case of a moderate infection of CAP, as far as absorption and pharmacokinetics allow. Furthermore, contraindications, severe penicillin allergy, and dose adjustment in kidney failure (after an adequate loading dose) should be considered. The proposal for the total duration of empirical antibiotic therapy was 5 days, with prolongation only in particularly justified cases (e.g., immunodeficiency or positive microbiological tests).

### Inclusion and exclusion criteria for the study

Adult (18 years or above) patients hospitalized due to CAP (based on ICD–International Classification of Disease code) who started their first empirical antibacterial therapy at the above-mentioned department were included in the study. All patients with CAP admitted from another hospital/ward having antibiotic therapy or coinfections at admission were excluded from the study.

### Data collection

All patient- and therapy-related data were collected manually by pharmacists. All data were assembled from medication charts and UD-MED Hospital Information System (IT Services, Hungary) and recorded in Microsoft Excel by using a predefined format of the data entity. Data collection forms were designed by the pharmacists. Demographics and clinical characteristics (gender, age, allergies, Charlson Comorbidity Index (CCI), weight, comorbidities, and discharge type), signs and symptoms (chills, fever, cough, dyspnea, chest pain, breathlessness, malaise, collapse, confusion, respiratory rate, blood pressure, presence of sputum, and dehydration) at admission, chest X-ray examination, laboratory test results (blood urea nitrogen (BUN) concentration, sodium, glucose, hematocrit, partial pressure of oxygen, respiratory rate, blood pressure, heart rate, pH, white blood cells (WBC), C-reactive protein (CRP), creatinine, estimated glomerular filtration rate (eGFR), lactase dehydrogenase enzyme (LDH), microbiological tests, and pathogens), empirical antibiotic therapy (agent selection, route of administration, dose, and duration), and clinical outcomes (30-day survival and length of stay (LOS)) were recorded on data collection forms. Empirical treatment is defined as the antibacterial therapy initiated based on the presence of suspected pathogens without any microbiological testing. Patients were anonymized, thus making them unidentifiable in the study.

### Pre-intervention and ASP period main outcome measures

The data obtained in both (pre-intervention phase and ASP phase) periods were analyzed and compared. We compared empirical antibiotic use regimen (types: mono- or combination therapy of the first empirical therapy, agent selection, dosage, and duration), the rate of guideline adherence, need for antibiotic change, antibiotic exposure (DDD/patient), antibiotic costs, as well as clinical outcomes (30-day survival and length of stay), and the need for antibiotic prescription at discharge.

Antibiotic exposure was determined using the World Health Organization’s ATC/DDD index (version 2023). The defined daily dose (DDD) is the assumed average maintenance dose per day for an agent used for its main indication in adults. Direct empirical antibiotic costs were calculated based on actual prices obtained from the central hospital pharmacy and expressed in HUF/patient. Antibiotic selection and duration of antibiotic use recommended for CAP are included in Table 5. One treatment followed by another was considered consecutive therapy. Any switch from an intravenous to oral regimen was considered sequential therapy. Empirical antibiotic therapy was considered guideline-adherent when all members were used appropriately regarding the severity of CAP. LOS indicated the number of days that the patient spent in the hospital.

The obtained data were compared to evaluate the effects of ASP implementation on empirical antibiotic use. We applied interrupted time-series analysis (ITSA) to express antibiotic prescription patterns in CAP in pre-intervention and ASP periods.

### Statistical analysis

Fisher’s exact test was applied to compare categorical variables, while Pearson’s correlation coefficient and *t*-test were used to compare continuous variables between the two study periods. *P* values below 0.05 were defined as significant.

## Results

In the pre-intervention and ASP periods, data from n = 131 and n = 227 patients, respectively, were collected, out of which data obtained from 78.6% to 85.5% patients met the study criteria and were included in the research. Included and excluded patients are mentioned in Table 2.

**TABLE 2:** Patients included and excluded from the study.

	Pre-intervention phase	ASP phase
Total number of hospitalized patients with CAP	N = 131 (100%)	N = 227 (100%)
Excluded patients	N = 28 (21.4%)	N = 33 (14.5%)
Admission from another ward/hospital	11 (8.4%)	3 (1.3%)
No antibiotic therapy	3 (2.3%)	4 (1.8%)
Targeted antibiotic therapy	2 (1.5%)	8 (3.5%)
Antibiotic therapy at admission	8 (6.1%)	13 (5.7%)
Coinfections	4 (3.1%)	5 (2.2%)
Included patients	N= 103 (78.6%)	N= 194 (85.5%)

ASP, antibiotic stewardship program; CAP, community-acquired pneumonia.

### Patient’s characteristics, signs, and symptoms

Demographic and clinical characteristics of patients hospitalized with CAP are described in Table 3. No significant differences were found regarding their gender, age, comorbidities, discharge types, outcomes, PSI, and CURB-65 scores between the two periods. In both periods, more than half of the patients (61–59.2% and 112–57.7%, respectively) were men, aged between 65 and 84 years, and had a CCI score above 4. The most common comorbidities included chronic obstructive pulmonary diseases and cardiovascular diseases. The majority of patients were discharged home. The 30-day mortality rates were 27.2% and 21.6%, with 21.4% and 19.1% being in-hospital deaths (Table 3).

**TABLE 3:** Demographic and clinical characteristics of patients with CAP in the pre-intervention and ASP periods.

Parameter	Pre-intervention period N = 103 (100%)	ASP period N = 194 (100%)	*P*-value
Gender (male)	61 (59.2%)	112 (57.7%)	*n.s*
Age			
20–64 years	28 (27.2%)	58 (29.9%)	*n.s*
65–84 years	65 (63.1%)	103 (53.1%)	*n.s*
≥85 years	10 (9.7%)	33 (17.0%)	*n.s*
CCI—Charlson Comorbidity Index			
0	1 (1.0%)	9 (4.6%)	*n.s*
1	3 (2.9%)	11 (5.7%)	*n.s*
2	13 (12.6%)	7 (3.6%)	<0.05*
3	16 (15.5%)	23 (11.9%)	*n.s*
4	10 (9.7%)	39 (20.1%)	*n.s*
>4	60 (58.3%)	105 (54.1%)	*n.s*
Comorbidities			
Chronic obstructive pulmonary disease	52 (50.5%)	93 (48.0%)	*n.s*
Cardiovascular disease	31 (30.1%)	80 (41.2%)	*n.s*
Dementia	19 (18.4%)	24 (12.4%)	*n.s*
Diabetes mellitus	16 (15.5%)	46 (23.7%)	*n.s*
Solid tumor			
Localized	15 (14.6%)	18 (9.3%)	*n.s*
Metastatic	13 (12.6%)	13 (6.7%)	*n.s*
Cerebrovascular accident or transient ischemic attack	10 (9.7%)	19 (9.8%)	*n.s*
Chronic liver/kidney disease (moderate to severe)	8 (7.8%)	25 (12.9%)	*n.s*
Peptic ulcer disease	3 (2.9%)	13 (6.7%)	*n.s*
Peripheral vascular disease	1 (1.0%)	10 (5.2%)	*n.s*
Hematologic malignant diseases	0 (0.0%)	2 (1.0%)	*n.s*
Discharge types			
Discharged home	72 (69.9%)	147 (75.8%)	*n.s*
Moved to another hospital ward	3 (2.9%)	2 (1.0%)	*n.s*
Intensive care unit (ICU)	0 (0.0%)	0 (0.0%)	*n.s*
Long-term care	6 (5.8%)	8 (4.1%)	*n.s*
Outcome			
In-hospital mortality	22 (21.4%)	37 (19.1%)	*n.s*
30-day mortality	28 (27.2%)	42 (21.6%)	*n.s*
PSI—Pneumonia Severity Index			
Class I. (point 0)	0 (0.0%)	0 (0.0%)	-
Class II. (points <70)	19 (18.4%)	40 (20.6%)	*n.s*
Class III. (points 71–90)	21 (20.4%)	39 (20.1%)	*n.s*
Class IV. (points 91–130)	47 (45.6%)	80 (41.2%)	*n.s*
Class V. (points >130)	16 (15.5%)	35 (18.0%)	*n.s*
CURB-65 severity score			
Low severity (points 0)	22 (21.4%)	45 (23.2%)	*n.s*
Moderate severity (points 1–2)	72 (69.9%)	129 (66.5%)	*n.s*
High severity (points 3–4)	9 (8.7%)	20 (10.3%)	*n.s*

ASP, antibiotic stewardship program; *n.s*., non-significant; *significant *p*-value: <0.05.

Signs and symptoms at admission are listed in Table 4. No significant differences were found regarding signs and symptoms between the two periods.

**TABLE 4:** Sign and symptoms at admission.

Sign and symptom at admission	Pre-intervention period N = 103	ASP period N = 194
Chills and fever	35 (34.0%)	59 (30.4%)
Cough	67 (65.0%)	125 (64.4%)
Dyspnea	64 (62.1%)	163 (84.0%)
Chest pain	31 (30.1%)	58 (29.9%)
Malaise	22 (21.4%)	45 (23.2%)
Pleural fluid at chest X-ray	35 (34.0%)	82 (42.3%)
Presence of sputum	46 (47.6%)	111 (57.2%)

### Empirical antibiotic therapy for CAP and ASP implementation

The characteristics of empirical antibiotic therapy in CAP and outcomes are summarized in Table 5. Combination therapy was used for CAP in both the periods in the vast majority of cases (pre-intervention period: 74.8% vs ASP period: 76.8%). In the ASP period, the guideline-adherent agent selection increased significantly by 35% (from 58.3% to 93.3%; *p* = 0.015). However, the overall (agent selection, route of administration, dosage, and duration) guideline adherence increased only by 6.2% (*p* > 0.05). The inappropriate use of clarithromycin and doxycycline decreased by 38.2% (pre-intervention period: 5.8% vs., ASP period: 3.6%; *p* > 0.05), while respiratory fluoroquinolones were not used in the ASP period. At the same time, significant decreases in the use of guideline non-adherence combinations with metronidazole (*p* < 0.05) were observed between the two periods. In both periods, overdosing was relatively high (7.8% and 8.2%, respectively), while underdosing was not frequent. In the ASP period, shorter-duration antibiotic therapy (˂7 days) was significantly more frequent (47.0% vs. 22.3%; *p* = 0.004), while the total duration of in-hospital antibiotic therapy decreased significantly by 16% (from median 8 to 6 days; *p* < 0.001). Moreover, the total duration of antibiotic therapy, including antibiotic prescriptions at discharge, decreased by 4.5% (from 10.74 ± 4.78, median 11 days to 9.87 ± 4.95, median 10 days; *p* > 0.05). Furthermore, in the ASP period, a significant increase in the number of consecutive therapies (by 13.2%, from 14.6% to 27.8%; *p* = 0.045) was observed, whereas switching from intravenous to oral route of administration resulted in an increase by 10.5% (from 3.9% to 14.4%; *p* = 0.010). However, in the majority of cases, there were no significant differences in changes between the first empirical therapy (80.6% and 72.2%, respectively) and escalation (14.6% and 11.3%; *p* > 0.05).

**TABLE 5:** Characteristics of empirical antibacterial therapy of CAP in the study periods.

Parameter	Pre-intervention period N = 103 (100%)	ASP period N = 194 (100%)	Increase /decrease %	*p*-value
Types of the first antibiotic therapy				
Monotherapy	26 (25.2%)	45 (23.2%)	−2.0%	*n.s*
Combination therapy	77 (74.8%)	149 (76.8%)	2.0%	*n.s*
Route of administration				
Solely iv	24 (23.3%)	33 (17.0%)	−6.3%	*n.s*
Solely po	9 (8.7%)	19 (9.8%)	1.1%	*n.s*
iv-po	70 (68.0%)	142 (73.2%)	5.2%	*n.s*
Guideline-adherent antibiotic	60 (58.3%)	181 (93.3%)	35.0%	<0.05*
Amoxicillin/clavulanic acid ± clarithromycin	37 (36.0%)	114 (58.8%)	22.8%	<0.05*
Ceftriaxone ± clarithromycin	0 (0.0%)	44 (22.7%)	22.7%	<0.001**
Cefepime ± clarithromycin	23 (22.3%)	1 (0.5%)	−21.8%	<0.001**
Ceftazidime ± clarithromycin	0 (0.0%)	1 (0.5%)	0.5%	*n.s*
Piperacillin/tazobactam ± clarithromycin	0 (0.0%)	16 (8.2%)	8.2%	<0.05*
Other*	0 (0.0%)	5 (2.6%)	2.6%	*n.s*
Guideline non-adherent antibiotic	43 (41.7%)	13 (6.7%)	−35.0%	<0.001**
Clarithromycin and doxycycline	6 (5.8%)	7 (3.6%)	−38.2%	*n.s*
RFQ^a^	4 (3.9%)	-	−100.0%	<0.05*
Beta-lactam + metronidazole	9 (8.7%)	6 (3.1%)	−64.4%	*n.s*
Guideline-adherent agent(s)	60 (58.3)	181 (93.3%)	35.0%	<0.05*
Guideline-adherent agent and route of administration	59 (57.3%)	166 (85.6%)	28.3%	<0.05*
Guideline-adherent agent, route of administration, and dose				
Appropriate dose	48 (46.6%)	149 (76.8%)	30.2%	<0.05*
Overdose^b^	8 (7.8%)	16 (8.2%)	0.4%	*n.s*
Underdose^c^	3 (2.9%)	1 (0.5%)	−2.4%	*n.s*
Guideline-adherent agent, route of administration, dose, and duration				
Appropriate (7–14 days)	17 (16.5%)	44 (22.7%)	6.2%	*n.s*
<7 days	23 (22.3%)	91 (47.0%)	24.7%	<0.05*
>14 days	8 (7.8%)	14 (7.2%)	−0.6%	*n.s*
Duration of the first empirical antibiotic therapy (mean ± SD, median days)	6.45 ± 3.06 (6)	5.25 ± 2.99 (5)	−13.4%	<0.001**
Duration of the total antibiotic therapy (mean ± SD, median days)	8.17 ± 4.06 (8)	6.35 ± 3.92 (6)	−16.0%	<0.001**
Duration of the total antibiotic therapy^d^ (mean ± SD, median days)	10.74 ± 4.78 (11)	9.87 ± 4.95 (10)	−4.5%	*n.s*
DDD/patient (mean ± SD, median)	19.89 ± 11.66 (18)	14.52 ± 9.55 (14)	−23.6%	<0.001**
Direct empirical antibiotic costs (HUF/patient)	19,334.10 ± 46,040.22	10,582.25 ± 11,124.98	−33.2%	<0.05*
Number of consecutive antibiotic therapies				
1	89 (86.4%)	140 (72.2%)	−14.2%	*n.s*
2–3	15 (14.6%)	54 (27.8%)	13.2%	<0.05*
Changes in the first empirical therapy				
Sequential antibiotic therapy**	4 (3.9%)	28 (14.4%)	10.5%	<0.05*
De-escalation	1 (1.0%)	6 (3.1%)	2.1%	*n.s*
Escalation	15 (14.6%)	22 (11.3%)	−3.3%	*n.s*
No change	83 (80.6%)	140 (72.2%)	−8.4%	*n.s*
Need for antibiotic prescription	51 (49.6%)	110 (56.7%)	7.1%	*n.s*
LOS (mean ± SD, median days)	8.85 ± 6.10 (8)	7.09 ± 5.84 (6)	−13.5%	<0.05*
30-day survival	75 (72.5%)	152 (78.4%)	5.9%	*n.s*

CAP—community-acquired pneumonia; ASP: antibiotic stewardship program; *n.s*: non-significant (*p* > 0.05); iv: intravenously; po: orally; RFQ: respiratory fluoroquinolone.

^a^ No severe penicillin allergy.

^b^ Compared to summary of product characteristics (SPC), due to lack of guideline-recommended dose or in case of low levels of the estimated glomerular filtration rate (eGFR) and serum creatinine level.

^c^ Due to body weight; SD: standard deviation.

^d^ Inclusive antibiotic prescription; DDD: defined daily dose based on pneumonia severity, and/or infectious disease specialist suggestion; * doxycycline, moxifloxacin, or carbapenems; **switch from IV to oral regimen; LOS: length of stay; *significant *p*-value: <0.05; **significant *p*-value: <0.001.

### Antibiotic exposure and costs

Antibiotic exposure in the ASP period decreased significantly by 23.6% (from 19.89 ± 11.66 to 14.52 ± 9.55 DDD/patient; *p* < 0.001). As expected, the decrease in the CAP duration led to a lower costs of direct antibiotic therapy after ASP implementation (by 33.2%, from 19,334.10 ± 46,040.22 to 10,582.25 ± 11,124.98 HUF/patient; *p* < 0.001) (Table 5).

**FIGURE 2:**
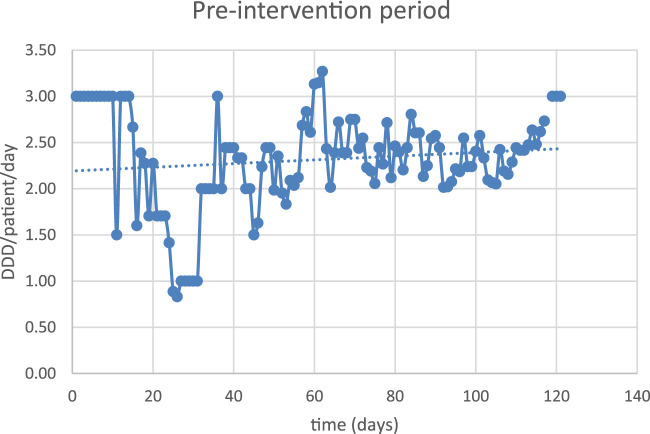
Antibiotic exposure in the pre-intervention period.

**FIGURE 3:**
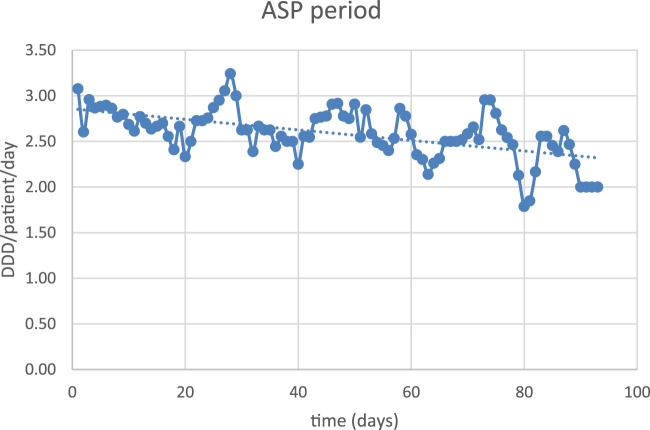
Antibiotic exposure in the ASP period.

The correlation coefficient between the two periods was −0.19 DDD/patient/day (95% CI –0.3831 to 0.0096), suggesting that the ASP has a sustained long-term effect (Figures 2, 3).

### Clinical outcomes: LOS and 30-day survival

In our study, comparing the pre-intervention period and ASP period revealed that the mean LOS decreased significantly by 13.5% (from 8.85 ± 6.10, median 8 days to 7.09 ± 5.84, median 6 days; *p* = 0.016). Guideline non-adherent combination therapies of beta-lactams with metronidazole were associated with prolonged LOS in both periods (pre-intervention period: 13.33 ± 9.79, median 11 days and ASP period: 9.5 ± 5.68, median 7 days) compared to the average LOS. Furthermore, we found that the 30-day survival rate increased by 5.9% (from 72.5% to 78.4%; *p* > 0.05) (Table 5).

## Discussion

Antimicrobial resistance continues to spread rapidly worldwide, threatening global public health (Allel et al., 2023). Antimicrobial stewardship programs (ASP) and local guidelines for empirical antibiotic therapies aim to slow the evolution of antibiotic resistance and improve clinical outcomes (WHO, 2021). CAP is still one of the most common acute infections requiring antibiotic therapy, and irresponsible antibiotic prescription may lead to overuse and misuse of these agents. The descriptions of guideline-adherent empirical antibiotic use in hospitalized patients with CAP vary widely (47.8%–65%) in the literature (Blasi et al., 2008; McCabe et al., 2009; Munther et al., 2023). In our previous study evaluating antibiotic prescription patterns, guideline adherence, and clinical outcomes in patients hospitalized with CAP (Fesus et al., 2022), there were no available local guidelines, and guideline adherence for agent selection was found to be low (30.6%). In contrast, in the pre-intervention period, when written guidelines were present on the ward, guideline adherence was found to be higher (58.3%).

Our healthcare center implemented its own strategy to optimize antibiotic use, in which physicians, infectious disease specialists, and clinical pharmacists had opportunities and responsibilities in optimizing empirical antibiotic therapies inclusive for CAP at the pulmonological department.

This study shows that in the pre-intervention period, the guideline non-adherent antibiotic use was relatively common (41.7%) for CAP in the pulmonological department. The quite frequent (8.7%) and redundant combinations of beta-lactams with metronidazole were associated with prolonged LOS.

In contrast, in the ASP period, the combination of the written local guideline and the restricted use of antibiotics resulted in significantly higher guideline adherence in agent selection (by 35%, from 58.3% to 93.3%) and remarkable improvement in LOS (by 13.5%, from mean 8.85 to 7.09 and median 8 to 6 days) in CAP. Although the 30-day survival also increased by 5.9% (from 72.5% to 78.4%), it was not statistically significant. Moreover, a significant improvement (by 30.2%, from 46.6% to 76.8%) in the appropriate use of antibiotics regarding agent selection, route of administration, and dosage was observed in the ASP period. Inappropriate use of metronidazole combination therapy was also decreased (by 64.4%, from 8.7% to 3.1%). Moreover, ASP led to a significant decrease in the total duration of antibiotic therapy (by 16.0%, from median 8 to 6 days), which in fact was associated with the significant decrease in direct empirical antibiotic costs. It should be noted that the sequential antibiotic therapy increased significantly by 10.5%. Although de-escalation and escalation also occurred, these efforts were not significant. At the same time, despite the lower antibiotic exposure and shorter duration of total empirical antibiotic therapy, the clinical outcomes (LOS) were improved (Table 5).

Based on the available evidence, it was found that ASP implementation was safe and led to benefits both for healthcare systems and patients (Viasus et al., 2017). In line with our results, the findings worldwide show that the ASP in CAP led to an increase in appropriate antibiotic therapy as well as a lower antibiotic exposure and duration of the antibiotic therapy (Markus Fally et al., 2020). A single-centered prospective pre- and post-intervention study showed an excessive decrease (from a median of 10 to 7 days, *p* ˂ 0.001) in the duration of antibiotic therapy with an ASP intervention (Avdic et al., 2012). In a study conducted at the Johns Hopkins Hospital among patients with CAP, the shorter-duration antibiotic therapy was shown to be as clinically effective as the longer-duration one (Nussenblatt et al., 2013). As indicated by our study results, half of the patients needed antibiotic prescriptions at discharge, and there was no difference between the two periods.

Several studies focused on de-escalation of empirical antibiotic therapy in CAP (Fally et al., 2021; Helen Umpleby et al., 2022; Waagsbo et al., 2022). According to a multi-center prospective study, the ASP resulted in a significantly higher rate of guideline-adherent antibiotic treatment and lower overall antibiotic exposure. However, there was no observed decrease in sequential therapy (Fally et al., 2021). At the same time, an observational study conducted in a teaching university hospital in Norway found that there was an existing need to complete the ASP for better antibiotic de-escalation strategies (Waagsbo et al., 2022). A narrative review evaluating the efficacy and risks of the ASP focused on de-escalation found that de-escalation is safe and encourages application of efforts also in this direction (Helen Umpleby et al., 2022). A cross-sectional study with the implemented ASP focused on decreasing the mean broad-spectrum days of the therapy, which yielded an absolute reduction of 1.7 days. The authors stated that a multifaceted ASP might safely reduce broad-spectrum antibiotic use (Schweitzer et al., 2022).

In line with the literature, in our study, the implementation of the ASP led to better clinical outcomes (LOS) and a significant decrease in direct empirical antibiotic therapy costs. A systematic review evaluating LOS, antibiotic exposure, and total costs after ASP implementation states that the highest cost savings came from scaling down of LOS, a fact observed mainly in those hospitals where the ASP included antibiotic restrictions as well (Nathwani et al., 2019).

## Strength and limitations

The manual data collection provided us first-hand observations on the antibiotic use in CAP at the pulmonological department. However, retrospective data collection from medical charts may contain inaccurate information or biases arising from inappropriate coding in electronic medical systems.

One of the most important limitations of the present study was that it is a single-center study involving a limited number of patients. The second limitation was that the study was conducted after 6 months of ASP implementation since in the pre-intervention period, due to the COVID pandemic, infectious disease specialists were redirected to infectious wards, and data collection was also obstructed. The ASP was implemented in January with the aim of collecting data according to the seasonal occurrence of CAP, and in June 2023, the CAP guidelines included in the ASP also underwent a few modifications.

## Conclusion

ASP implementation led to a significant improvement in overall guideline adherence, appropriate antibiotic use, sequential therapy, and a significant reduction in the total duration of empirical antibiotic therapy. The ASP was accompanied by a significant decrease in the hospital length of stay. Our study result suggests that the ASP may play an important role in optimizing empirical antibiotic therapy in CAP with a sustained long-term effect.

## Data Availability

The original contributions presented in the study are included in the article/Supplementary Material; further inquiries can be directed to the corresponding author.
